# The potential impact of Covid-19 on the capacity of routine laboratory tests to detect heparin-induced thrombocytopenia

**DOI:** 10.1186/s12959-022-00411-0

**Published:** 2022-09-26

**Authors:** Dominik F. Draxler, Justine Brodard, Björn Zante, Stephan M. Jakob, Jan Wiegand, Johanna A. Kremer Hovinga, Anne Angelillo-Scherrer, Alicia Rovo

**Affiliations:** 1 Department of Hematology and Central Hematology Laboratory, Inselspital, Bern University Hospital, University of Bern, Bern, Switzerland; 2 Department of Cardiology, Inselspital, Bern University Hospital, University of Bern, Bern, Switzerland; 3 Bern Center for Precision Medicine, Bern, Switzerland; 4 Department of Intensive Care Medicine, Inselspital, Bern University Hospital, University of Bern, Bern, Switzerland; 5 Department of Intensive Care Medicine, Lindenhofspital, Bern, Switzerland

**Keywords:** Covid-19, Heparin-induced thrombocytopenia, Heparin-induced platelet activation test

## Abstract

In Covid-19, anticoagulation with heparin is often administered to prevent or treat thromboembolic events. Heparin-induced thrombocytopenia (HIT) is a severe complication of heparin treatment, caused by heparin-dependent, platelet activating anti-platelet factor 4 (PF4)/heparin antibodies. Diagnosis of HIT is based on the combination of clinical parameters, allowing to determine the pretest probability, and laboratory testing for anti-PF4/heparin antibodies and confirmatory functional assays, such as the heparin-induced platelet activation (HIPA) test.

We report the case of a patient with severe Covid-19 pneumonia requiring ECMO treatment, who developed recurrent clotting of the ECMO filter and a drop in platelet count under heparin treatment. He was therefore suspected to have HIT and the anticoagulation was switched to argatroban. Despite high clinical probability and high titres of anti-PF4/heparin antibodies, the functional HIPA test was negative. Nevertheless, argatroban was continued rather than to reinstate anticoagulation with heparin. Reevaluation 7 days later then demonstrated a strongly positive functional HIPA test and confirmed the diagnosis of HIT. Under anticoagulation with argatroban the patient gradually improved and was finally weaned off the ECMO.

In conclusion, this case highlights the critical importance of clinical judgement, exploiting the 4 T score, given that Covid-19 patients may present a different pattern of routine laboratory test results in HIT diagnostics. The possibility of a false negative HIPA test has to be considered, particularly in early phases of presentation. In cases of a discrepancy with high clinical probability of HIT and/or high titre anti-PF4/heparin antibodies despite a negative HIPA test, a reevaluation within 3 to 5 days after the initial test should be considered in order to avoid precipitant reestablishment of unfractionated heparin, with potentially fatal consequences.

## Background

In Covid-19, anticoagulation with heparin is often administered to prevent or treat thromboembolic complications [1]. HIT is a severe complication of heparin treatment, caused by heparin-dependent, platelet-activating anti-PF4/heparin antibodies and carries a high risk for thrombotic events. HIT diagnosis is based on the combination of clinical and laboratory parameters. The 4 T score (which involves the criteria Thrombocytopenia, Timing, Thrombosis, and oTher causes, summarised in Table 1) determines the pretest probability of HIT [2]. Anti-PF4/heparin antibodies are then assessed by immunological assays and functional tests performed to demonstrate that anti-PF4/heparin antibodies are platelet-activating, thereby confirming the diagnosis HIT.

**Table 1:** Calculation of the 4 T Score

	Points (0, 1, or 2 for each of the 4 categories: Maximum Possible Score = 8)		
	2	1	0
Pretest probability score: 6–8 indicates high; 4–5, intermediate; and 0–3, low			
^a^First day of immunizing heparin exposure considered day 0			
Thrombocytopenia	** > 50% platelet fall to nadir ≥ 20 G/l**	30–50% platelet fall, or nadir 10–19 G/l	< 30% platelet fall, or nadir < 10
Timing^a^ of onset of platelet fall (or other sequelae of HIT)	Days 5–10, or ≤ day 1 with recent heparin (past 30 days)	** > Day 10 or timing unclear; or < day 1 with recent heparin (past 31–100 days)**	< Day 4 (no recent heparin)
Thrombosis or other sequelae	**Proven new thrombosis; skin necrosis; or acute systemic reaction after intravenous UFH bolus**	Progressive or recurrent thrombosis; erythematous skin lesions; suspected thrombosis (not proven)	None
Other cause(s) of platelet fall	None evident	**Possible**	Definitive

Pretest probability score: 6–8: high; 4–5: intermediate; 0–3: low

Selected criteria in the presented case in bold, resulting in a score of 6/8 points

^a^First day of immunising heparin exposure considered day 0

While the incidence of HIT is surprisingly low in intensive care unit (ICU) populations [3], a significant incidence in patients on extracorporeal membrane oxygenation (ECMO) has been reported [4]. More recently, case reports have described the development of HIT in patients with severe Covid-19 pneumonia, particularly when treated with ECMO [5, 6].

Given the nowadays well-accepted interaction of Covid-19 and routine laboratory diagnostics for HIT [7], we here report the case of a patient with severe Covid-19 pneumonia requiring veno-venous (vv) ECMO treatment. Based on initial functional laboratory testing, HIT would have been deemed unlikely despite being present. While the immunological anti-PF4/heparin antibody assay was strongly positive early on, the first confirmatory functional assay performed was negative and became only positive in the second assay, which was performed 7 days after the first one. This case highlights the critical importance of clinical judgement, given that Covid-19 patients may present a different pattern of routine laboratory test results in HIT diagnostics [8].

## Case presentation

A 67-years old Caucasian male patient was admitted to the ICU of our tertiary care institution in January 2021 with severe acute respiratory distress syndrome (ARDS) due to a Covid-19 pneumonia. The patient had a known history of non-insulin dependent diabetes mellitus type 2 treated with oral antidiabetics, of arterial hypertension and obesity class 1.

He had initially been hospitalised at a peripheral secondary care centre because of progressive dyspnoea, and was intubated in the further course due to respiratory deterioration. In addition, a therapy with dexamethasone and therapeutic anticoagulation with unfractionated heparin was initiated. Because of the development of a ventilator-associated pneumonia (VAP), antibiotic therapy with piperacillin and tazobactam was started, which was later escalated to meropenem, vancomycin and fluconazole due to a suspected bloodstream infection. Later, evidence of Staphylococcus epidermidis and Staphylococcus capitis were found in cultures derived from the tip of the removed central venous line. A tracheotomy was performed when gas exchange and lung compliance progressively deteriorated. Seventeen days after admission, the patient was transferred to the ICU in our hospital for insertion of a vv-ECMO and dexamethasone was discontinued. On day 2 after admission to our ICU, a high-dose steroid therapy (methylprednisolone 160 mg daily) was initiated.

Recurrent clotting of the ECMO filter and a drop in platelet count was observed during the first 24 h of ECMO treatment. A 4 T score of 6 out of 8 points suggested high clinical probability of HIT (Table 2). The diagnosis was further enforced by a positive HIT laboratory test, determining the levels of anti-PF4/heparin antibodies in plasma samples, using an anti-PF4/heparin ELISA (PF4 IgG GTI ELISA, Immuncor, Waukesha, USA) [9]. In addition, a functionally confirmatory HIPA test, assessing platelet aggregation in the presence and absence of heparin, was initiated. The detailed method of this assay as performed at our institution is described elsewhere [10]. Platelets used for this functional test are obtained from the from the local blood donation service (SRK Bern, Blutspende Dienst).

**Table 2:** Relevant clinical and laboratory parameters assessed in the course of this case

Clinical/laboratory parameter	Day 1 of suspected HIT	Day 8 of suspected HIT	Test interpretation
4 T score	6/8	-	0–3 low probability 4–5 intermediate probability 6–8 high probability
HIT-IgG Acustar (U/ml)	10.8	43.2	< 1.0 U/ml
HIT ELISA IgG (O.D.) (Immuncor, Waukesha, USA)	1.465	2.902	< 0.4 negative for anti-PF4 antibodies 0.4–1.0 weak positive for anti-PF4 antibodies > 1.0 positive for anti-PF4 antibodies
HIPA (wells + out of 4)	1/4	4/4	≤ 1/4 wells negative for HIT ≥ 2/4 wells positive for HIT
HIPA interpretation	negative	positive	-

*ELISA* Enzyme-linked immunosorbent assay, *HIPA* Heparin-induced platelet activation test, *HIT* Heparin-induced thrombocytopenia, *O.D.* Optical density

Unfractionated heparin was discontinued and switched to argatroban, a direct thrombin inhibitor, as an alternative anticoagulant for patients with suspected HIT, and the ECMO system was exchanged. A diffuse haemoglobin-relevant bleeding from the ECMO cannula occurred from the right jugular vein. One packed red blood cell concentrate was administered and the bleeding ceased spontaneously in the further course without interruption of the therapeutic anticoagulation with argatroban [10].

Interestingly, the initial HIPA test showing heparin-dependent platelet aggregation in only 1 out of 4 wells later suggested absence of HIT. However, due to the high clinical probability of HIT (based on the 4 T score) in this particular patient, we decided not to switch from argatroban back to heparin immediately, but rather to maintain anticoagulation with argatroban and to repeat the HIPA test for confirmation. The second HIPA test was performed 7 days after the first one. In this second HIPA assay, 4 out of 4 wells were positive for platelet aggregation and therefore the test strongly confirmed the presence of HIT (Table 2). Consequently, the anticoagulation with argatroban was continued and exposure to heparin further avoided. Regarding other differential diagnoses, we also considered a disseminated intravascular coagulation due to a drop in fibrinogen levels and impaired plasmatic coagulation, which was soon ruled out based on the favourable clinical development. Recurrent clot formation continued to occur in the ECMO system, but without compromised flow. No further thrombotic complications were observed, although platelet counts remained at a low level.

**Fig. 1:**
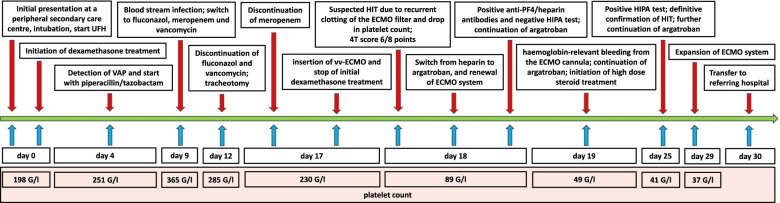
Summary of the key events with respect to ICU treatment and HIT diagnostics. Abbreviations: ECMO, extracorporeal membrane oxygenation; HIPA, heparin-induced platelet activation test; HIT, heparin-induced thrombocytopenia; UFH, unfractionated heparin; VAP, ventilator-associated pneumonia

The patient was deeply sedated and muscle relaxants were used to enable relaxation for protective ventilation. Starting 2 days after admission to our ICU and ECMO insertion (day 19 after intubation), we were able to slowly reduce the sedation. An opiate rotation with methadone was performed and the patient could be gradually weaned off the vv-ECMO. On day 30, the patient was hemodynamically stable without vasoactive agents, and was transferred to the referring institution for further treatment, and afterwards to rehabilitation for five weeks. At the time of discharge from our ICU, the patient was continued on argatroban and methylprednisolone 160 mg daily with the aim to reduce the steroid dose in the further course. The patient is alive and in clinical follow-up for Covid-19 sequelae. The most important events with respect to HIT diagnosis and treatment during his hospitalisation are summarised in Fig. 1.

## Discussion

Covid-19 is associated with a prothrombotic state [11–14] and has been reported to induce venous thromboembolism in up to 30% of severe cases [15]. The underlying pathophysiologic mechanism for this phenomenon is complex and appears to involve dysregulation of both cellular and plasmatic coagulation, as well as fibrinolysis [16–19]. In severe Covid-19, thrombus formation is driven by a cytokine storm and inflammation, as well as activation of the complement system, platelets, endothelial cells, and microvesicles [20]. Further prognostic factors for thrombotic events include age over 60 years, hypertension, diabetes and D-dimer levels > 3.17 µg/ml [21]. Thromboprophylaxis with low molecular weight heparin nowadays represents part of the standard treatment in severely ill patients with COVID-19 [1]. Importantly, the presence of anti-PF4/heparin antibodies has been reported in Covid-19, unrelated to previous exposure to heparin and the presence of HIT [7, 8].

Anti-PF4/heparin antibodies are used as a diagnostic tool for HIT, in addition to the assessment of the clinical probability based on the 4 T score. Nevertheless, while frequently used as the only laboratory parameter for HIT assessment, the presence of anti-PF4/heparin antibodies is not sufficient to confirm HIT diagnosis. For a definitive diagnosis, functional assays confirming the anti-PF4/heparin antibodies’ capacity to induce platelet activation/aggregation, such as the HIPA test have to be utilized [22, 23]. In published investigations, there is significant variability in the way HIT has been identified. While some studies used merely clinical criteria, others determined anti-PF4/heparin antibodies, with only some confirming their platelet-activating properties in functional tests [7].

The overall incidence of HIT in patients exposed to heparin has been estimated to be 0.2%—5% [24, 25], with surprisingly low numbers (0.5%) in ICU populations [3]. However, an incidence of up to 7.3% has been reported in patients on ECMO [4]. Parzy et al. investigated 13 severe ARDS cases associated with Covid-19, all requiring a vv-ECMO, and all developing venous thromboembolism [26]. Three out of these 13 reported cases (23.1%) had a HIT confirmed by laboratory testing, although the test method was not indicated in the study. Bidar et al. presented the cases of 2 patients with severe Covid-19 pneumonia who required vv-ECMO and developed HIT. In these cases HIT was diagnosed by elevated anti-PF4/heparin antibody levels and confirmed with HIPA. The authors concluded, that argatroban as an alternative anticoagulation to heparin was effective and safe in Covid-19 patients on vv-ECMO with HIT [5]. These findings were similar to those previously published in another case report on a Covid-19 patient on veno-arterial ECMO by Ogawa et al. [6].

In the presented case, both the 4 T score and the levels of anti-PF4/heparin antibodies were highly suggestive of HIT, however the initial HIPA test was negative. This particular constellation of laboratory parameters has been previously reported in Covid-19 patients with clinically suspected HIT [8]. It was observed that Covid-19 patients present with high levels of anti-PF4/heparin antibodies in the context of the infection, resulting in reduced specificity of this parameter for the detection of HIT with frequent false positive test results. In the case we present here, the clinical probability of HIT was high and we decided to continue anticoagulation with argatroban despite the negative HIPA test. The repetition of HIT evaluation was performed 7 days after the first assay and the result of this second HIPA assay was clearly positive, confirming the diagnosis of HIT and supporting the need to continue anticoagulation with argatroban. It has to be highlighted here as a methodological limitation, that the set of donors for this second round was not the same as for the first round, given that blood samples are always freshly obtained and the donors therefore vary between runs. Nevertheless, the use of positive and negative controls for the assay [10] preclude that the initial negative response was due to unresponsive blood donors. Genetic polymorphisms of the FcRIIa receptor (i.e. Arg/His131 and Gln/Lys127) which are rare in the general population can induce a lack of response in the HIT functional assay [27]. This is the reason why the laboratory must have the availability of a supplemental donor sample as backup.

Supporting the critical role of clinical judgement in cases like this, Favaloro et al. highlighted in their recent review, that there was a higher 4 T score (median 4 T score 6/8 points) in individuals with Covid-19, who had a positive functional HIT test, as compared with those with a negative functional HIT test (median 4 T score 4/8 points) [7]. While the authors acknowledge the low number of studies, this still somewhat confirms the substantial value of the 4 T score also in COVID-19 patients with suspected HIT.

This case highlights that high clinical probability for HIT, assessed with the 4 T score, is indeed a strong indicator for the presence of HIT also in Covid-19 patients. In such cases, all negative test results should be critically interpreted, particularly in the early phase, and might have to be repeated. While elevated anti-PF4/heparin antibodies appear not to be a reliable indicator for the presence of HIT in Covid-19 patients [8], the functional HIPA test is a valuable diagnostic tool to confirm the diagnosis, yet must be repeated before definitively ruling out the diagnosis of HIT 3 to 5 days after the initial test, in cases, where high clinical probability is accompanied by a negative test result. The exact mechanistic explanation for this phenomenon remains elusive, yet our observation indicates, that the development of specific platelet-activating antibodies may take a few days, so that a robust positive HIPA test result may only be detectable several days after presentation of the initial clinical features of HIT (i.e. thrombosis). This should be considered in patients with Covid-19 and a high probability for HIT, in order to avoid a rushed discontinuation of non-heparin anticoagulation and reestablishment of heparin, which may have catastrophic consequences for patients in need of appropriate anticoagulation. Whether our observation is related to the Covid-19 infection itself, or can be explained by other individual features of this particular patient remains unclear. In this regard, it has to be highlighted as well, that the secondary bacterial infection may be an additional pathomechanistic trigger for the development of HIT, as previously described in a review article by Root-Bernstein [28]. In addition, it remains unclear, whether our observation of delayed responsiveness is restricted to the HIPA test, or can also be observed in other functional assays used to confirm the diagnosis of HIT, such as the serotonin release assay. In our opinion, caution should be taken, and the functional test repeated, regardless of the assay used, if doubt about an early negative result exists, based on the clinical probability.

In conclusion, clinical judgement in HIT diagnostics, harnessing the 4 T score is crucial, considering the possibility of a false negative HIPA test, particularly in early phases of presentation. In cases of a discrepancy with high clinical probability of HIT despite a negative HIPA test, a repetition of the HIPA test should be considered in order to avoid precipitant reestablishment of unfractionated heparin, with potentially fatal consequences.

## Data Availability

Not applicable.

## Abbreviations

ARDS: Acute respiratory distress syndrome
ECMO: Extracorporeal membrane oxygenation
HIT: Heparin-induced thrombocytopenia
HIPA: Heparin-induced platelet activation test
ICU: Intensive care unit
PF4: Platelet factor 4
VAP: Ventilator-associated pneumonia
vv: Venous-venous
